# The therapeutic effect of miR-125b is enhanced by the prostaglandin endoperoxide synthase 2/cyclooxygenase 2 blockade and hampers ETS1 in the context of the microenvironment of bone metastasis

**DOI:** 10.1038/s41419-018-0499-8

**Published:** 2018-04-27

**Authors:** Paola Maroni, Paola Bendinelli, Emanuela Matteucci, Maria Alfonsina Desiderio

**Affiliations:** 1 Istituto Ortopedico Galeazzi IRCCS, Via R. Galeazzi 4, 20161 Milano, Italy; 2 Dipartimento di Scienze Biomediche per la Salute, Molecular Pathology Laboratory, Università degli Studi di Milano, Via L. Mangiagalli 31, 20133 Milano, Italy

## Abstract

Bone is the most common site for breast cancer spread. In the pro-metastatic cell line 1833, derived from MDA-MB-231 breast adenocarcinoma cells, both hypoxia and hepatocyte growth factor (HGF) influence the effect of miR-125b on ETS proto-oncogene 1 transcription factor (ETS1). The effect of hypoxia inducible factor 1 alpha subunit (HIF1A), known to promote metastatic spread by upregulating prostaglandin endoperoxide synthase 2 (PTGS2), may be dampened by miR-125b targeting PTGS2. Here, we investigated whether miR-125b plays a role in breast cancer metastasis by measuring its activity in response to the chemotherapeutic agent NS-398 in a xenograft model. NS-398 is typically used in the clinic to target PTGS2. We also aimed to describe the molecular mechanisms in vitro, since the enhancement of epithelial properties may favor the efficacy of therapies. We report that in the xenograft model, miR-125b reduced metastasis to the bone. We also report suppression of PTGS2 enhanced survival by decreasing HIF1A in cells within the bone marrow. In 1833 cells transfected with a miR-125b mimic we observed several phenotypic changes including enhancement of the epithelial marker E-cadherin, a reduction of mesenchymal-associated genes and a reduction of WNT-associated stem cell signaling. Our findings suggest that in vivo, key players of the bone microenvironment promoting breast cancer spread are regulated by miR-125b. In future, biological molecules imitating miR-125b may enhance the sensitivity of chemotherapeutic agents used to counteract bone metastases.

## Introduction

Disseminated tumor cells (DTC) from breast carcinoma coordinate the formation of a favorable microenvironment for future bone metastases, and the bone marrow is implicated through growth factors and various cellular types of the staminal hematopoietic and osteoblastic niches^1,2^. The presence of micrometastases, within the bone marrow premetastatic niche, is used as a predictive index for the survival of breast carcinoma patients; micrometastasis evolution leads to osteolytic metastases, involving the osteoclast-mediated reactivation of dormant metastatic cells^3,4^. Xenograft models are suitable to examine the molecular modifications in metastatic cells, supportive cells and extracellular matrix (ECM), which underlie the interaction between metastasis and stroma. Novel therapeutic approaches might counteract the favorable milieu of components in diseased bone after the DTC access^5,6^.

Microenvironmental stimuli contribute to the phenotype plasticity, which is critical for the metastatic process of DTC engrafted in the skeleton^6–9^. In human bone metastasis specimens, the hepatocyte growth factor (HGF)/Met receptor signaling pathway correlates with miR-34a down-regulation^10^. Since miRNAs play a role in mRNA degradation or decrease of protein expression, and this is consequence of the binding to the 3′-untranslated region (3′UTR) (post-transcriptional level)^11,12^, miR-34a seems to target Met in skeleton metastases but not in the pair-matched primary breast carcinomas^10^.

The knowledge of miRNA functions in bone metastasis phenotype would be important for therapeutic purpose^13,14^. miRNA deregulation seems to depend on the microenvironment stimuli and/or on epigenetic control, and miRNA changes might inhibit or induce the epithelial phenotype affecting invasion and metastasis^15–17^. The weakening of tumor cells adhesion, and the strengthening of cell motility and proteolysis trigger epithelial–mesenchymal transition (EMT) and invasion. Notably, the mesenchymal phenotype and stemness are associated with neoplasia resistance to therapy^7,18–20^.

The miR-125b suppresses the osteogenic differentiation of mesenchymal stem cells derived from bone marrow^21^, and it targets specific signaling pathways such as those regulated by ETS proto-oncogene 1 transcription factor (ETS1)^13^. In 1833 bone metastatic cells, derived from MDA-MB-231 breast adenocarcinoma cells, the effects of miR-125b on ETS1 activity and on the biological functions are influenced by HGF and hypoxia, which are stimuli of the bone microenvironment^22^. miR-125b acting as a tumor suppressor may translationally repress Human antigen R (HuR), which affects the stability and translational efficiency of target mRNAs^11^. Thus, the expression of genes such as prostaglandin endoperoxide synthase 2 (PTGS2) (previously known as cyclooxygenase 2), showing three HuR binding sequences in the 3′UTR^11^, might be controlled by miR-125b. PTGS2 plays multiple roles in primary tumors and bone metastases^23,24^. In 1833 cells, PTGS2 is implicated in the nuclear translocation of hypoxia inducible factor 1 alpha subunit (HIF1A) and in HIF1 activation and, therefore, in angiogenesis important for metastasis outgrowth^25^. Also, miR-125b seems to reverse multidrug resistance by modulating anti-apoptotic factors^26^. Even if some miRNA roles in cancer treatment have been clarified^27^, pre-clinical studies for antimetastatic therapies based on miRNA mimic are still lacking.

The aim of the present paper is to provide the molecular basis for the design of novel combined therapies to switch metastatic cells towards the epithelial phenotype, reducing also stem cell properties: this tool would permit to enhance the sensitivity to chemotherapeutic agents. Thus, the xenograft model of bone metastasis was prepared with 1833 cells transfected with miR-125b mimic, and the PTGS2 inhibitor N-(2-cyclohexyloxy-4-nitrophenyl)methanesulfonamide (NS-398) was administered to the mice^28^. miR-125b mimic might hamper bone metastasis colonization, by down-regulating ETS1 and probably by affecting indirectly epithelial and stemness genes^11,13,29^, and might enhance the inhibitory effect of anti-PTGS2 chemotherapy. We studied E-cadherin, which is a marker of epithelial phenotype of skeleton metastasis from breast carcinoma^18,19,30^, while the mesenchymal markers examined were matrix metalloproteinase2 (MMP2), essential for dissemination of metastatic cells by remodeling ECM (basal membrane and interstitial matrix), and vimentin which promotes adhesion/motility^19,20^. The WNT pathway activity would indicate the acquirement of stem cell properties, and it is targeted by miRNAs downstream of ETS1^18,29^. We expect that miR-125b might interrupt the interaction between ETS1 and HIF1A^22^, impairing directly and/or indirectly PTGS2, which is a target gene of HIF1^25^. ETS1 acts as a player for the invasive program depending on the conditions of the osseous microenvironment^22,31^.

Under miR-125b and NS-398 combined treatment, we observed the reduction and delay of bone metastasis outgrowth, and decreases of the expression of ETS1, HIF1A and PTGS2 together with phenotypic changes. In vitro, hypoxia and HGF interfered in the changes of gene expression due to miR-125b.

## Results

### Effect of miR-125b and NS-398 on bone metastasis growth

**Fig. 1:**
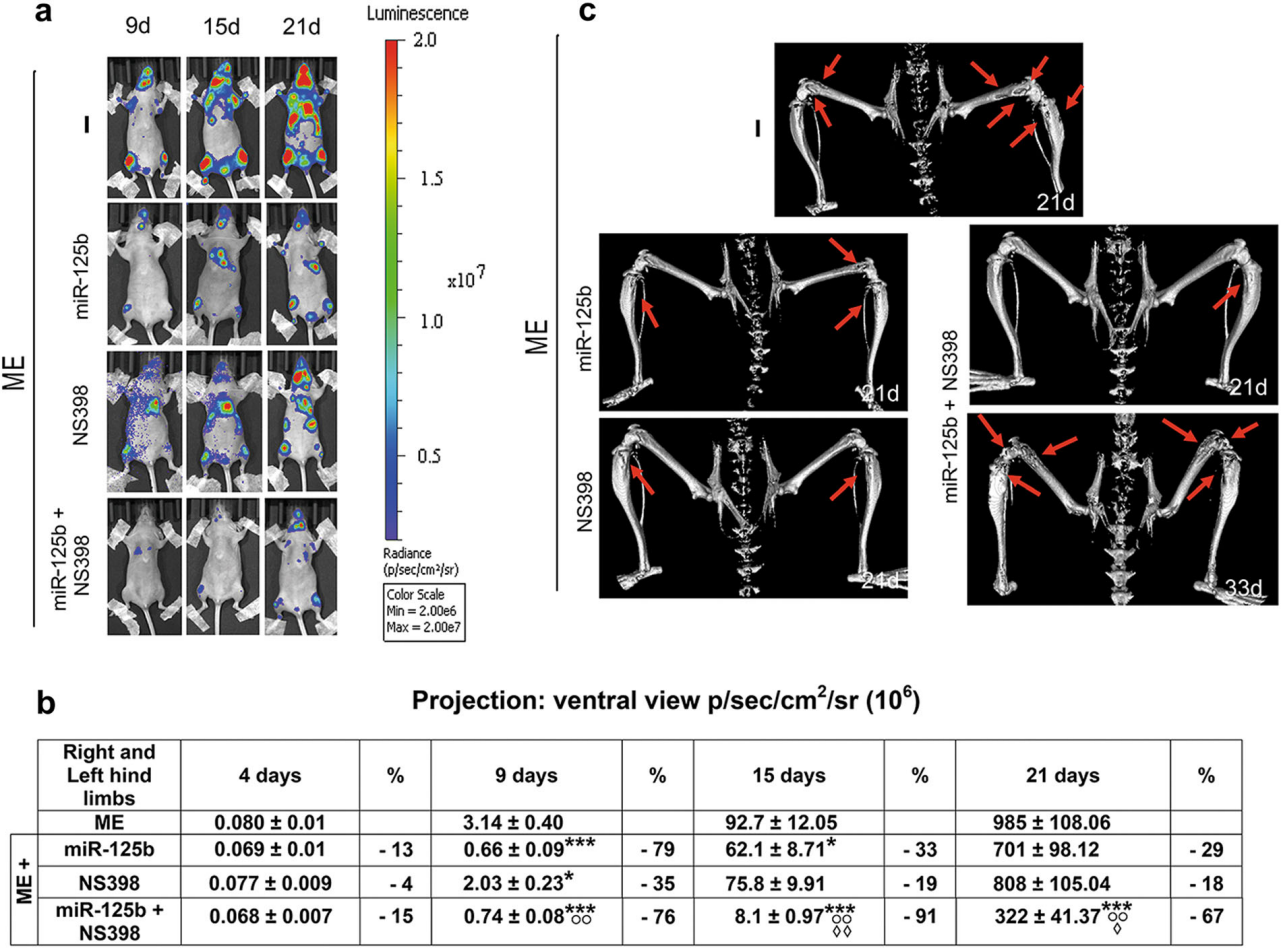
miR-125b alone or in combination with NS-398 affected bone metastasis formation in the xenograft mice. **a** Representative bioluminescence imaging (BLI) of the xenograft mice treated as shown. ME, mice bearing bone metastases. **b** We report quantitative data for the bioluminescence signals, and the percent of inhibition due the treatments vs. ME. The data are the means ± S.E. of five mice per group. *P < 0.05, ***P < 0.001 vs. the bioluminescence value of ME at the corresponding time; °°*P* < 0.005 vs. the value under NS-398 treatment at the corresponding time; ^◊^*P* < 0.05, ^◊◊^*P* < 0.005 vs. the value under miR-125b at the corresponding time. **c** Representative images of the three-dimensional reconstruction of μCT at 21 and 33 days of treatment. Three xenograft mice per group were analyzed with similar results. The arrows indicate osteolytic lesions

**Fig. 2:**
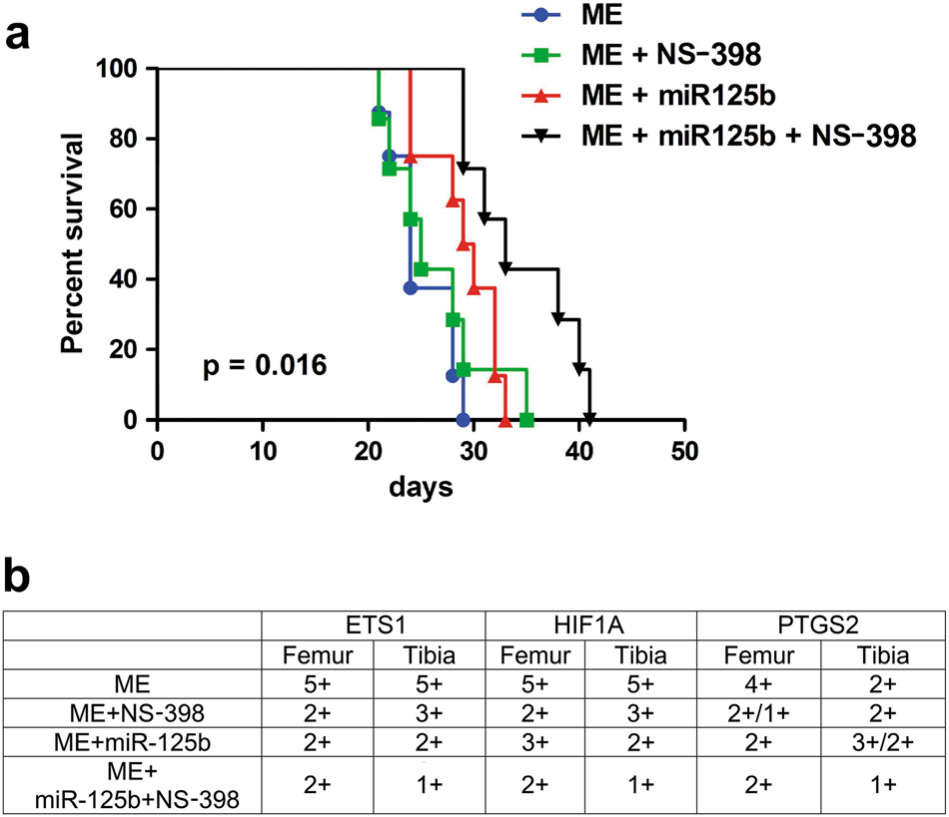
Effect of miR-125b, NS-398 and their combination on xenograft mice survival, and on gene expression in bone metastasis. **a** Survival curve on Kaplan-Meier plots of the data of treated and untreated ME. **b** Semi-quantitative evaluation of immunohistochemistry assays of ETS1, HIF1A and PTGS2 in bone metastasis of xenograft mice treated as indicated. We analyzed serial sections for each specimen from three mice per group of treatments at 26 days, and we found similar results

Considering that miR-125b targets a wide spectrum of genes including PTGS2 and ETS1, and that miRNAs are proposed as tools for cancer therapy, we tested miR-125b alone or in combination with NS-398, a specific chemical inhibitor of PTGS2^28^. In Figs. 1 and 2, we evaluated the growth of bone metastasis and the survival of the xenograft mice under these treatments. To prepare the xenograft model, we used 1833-bone metastatic cells engineered with the triple reporter construct with luminescence (1833/TGL)^32^.

As shown in Fig. 1a, we performed the time-course of bioluminescence images to evaluate the bone-metastasis development after 1833/TGL injection (ME): ME was the control mice group, and the effects of the single treatments or their combination were evaluated in respect to ME. Similar bioluminescence signals were observed for ME and for the xenograft mice injected with miR-neg-CTR cells (data not shown).

After quantification of the bioluminescence, the values for the hind limbs were reported in Fig. 1b. All the treatments did not practically affect metastatic growth within 4 days from 1833/TGL injection. At 9 days, miR-125b alone or in combination with NS-398 markedly prevented metastasis development, in respect to ME and to NS-398 alone. Importantly, only for the combination miR-125b plus NS-398 this strong inhibitory effect persisted until 21 days.

In addition, we demonstrated by micro-computed tomography (μCT) analysis of the hind limbs that miR-125b plus NS-398 was effective in preventing at a great extent the osteolytic lesions at 21 days, making the comparison with ME at the same time (Fig. 1c). The single treatments significantly reduced osteolysis at 21 days in the hind limbs. Of note, in the mice treated with miR-125b plus NS-398 the osteolytic lesions were evidenced again at 33 days (Fig. 1c). Consistently, the metastasis wideness was less at 26 days than at 29 days, and the bioluminescence signal at 26 days was reduced of about 60% by the combined treatment (Supplementary Figure S1).

Figure 2a shows that the exposure to miR-125b plus NS-398 prolonged the mice survival of about 12 days in respect to that of ME, and the life-protective effect of the therapy combination was evident also in comparison with the single treatments.

Based on these data, we examined by immunohistochemistry at 26 days the expression of the target genes of miR-125b as well as of HIF1A, known to be implicated in PTGS2 regulation via HIF1 activity (Fig. 2b)^25^. A semiquantitative evaluation of the immunohistochemistry data was performed, considering low (2+), mild (3+), high (4+) and very high (5+) signals. The expression of ETS1 and HIF1A was more elevated than that of PTGS2 in ME-bone metastases: these gene signals were strongly reduced (from 50 to 80%) by the combined exposure to miR-125b plus NS-398, while the single treatment miR-125b or NS-398 seemed less effective.

Altogether, our findings suggested a key role of the genes examined in bone metastasis growth, and the efficacy of the combined treatment tested.

### Expression of ETS1, HIF1A and PTGS2 and their intracellular distribution in bone metastasis under miR-125b plus NS-398

**Fig. 3:**
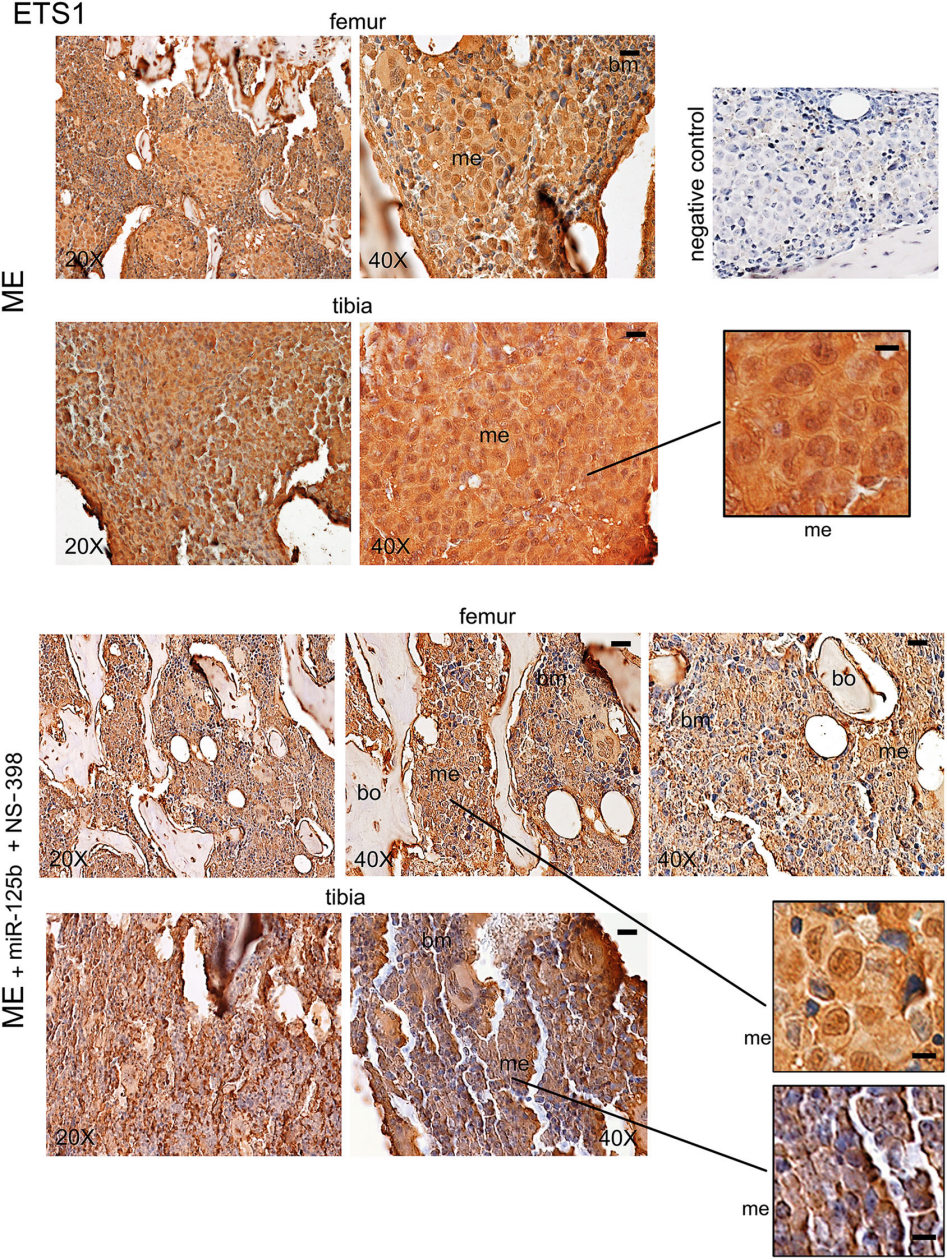
miR-125b in combination with NS-398 affected ETS1 expression in bone metastasis of xenograft mice. We show representative images of ETS1 in bone metastasis from mice treated with the combination miR-125b plus NS-398, or not (ME). Serial sections for each specimen from three mice at 26 days were analyzed obtaining similar results. me, metastasis; bm, bone marrow; bo, bone. Scale bar = 120 μm (reported in 40× magnification panels). Scale bar = 60 μm (reported in the three insets). The negative control was performed without the specific antibody

**Fig. 4:**
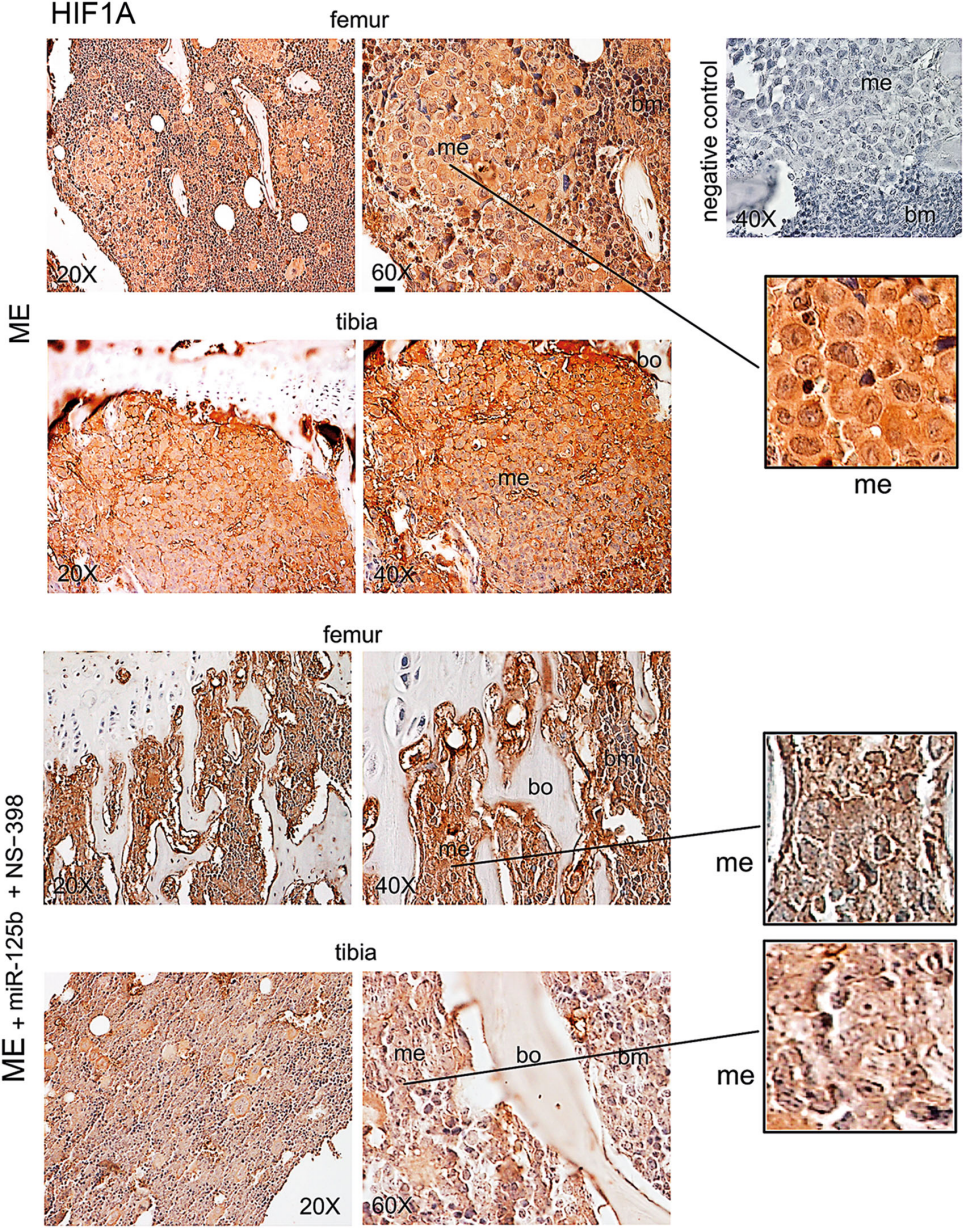
HIF1A expression in bone metastasis of xenograft mice under miR-125b in combination with NS-398. We show representative images of HIF1A in bone metastasis of mice treated with the combination miR-125b plus NS-398, or not (ME). Serial sections for each specimen from three mice at 26 days were analyzed obtaining similar results. me, metastasis; bm, bone marrow; bo, bone. Scale bar = 120 μm (reported in an exemplificative panel). The negative control was performed without the specific antibody

**Fig. 5:**
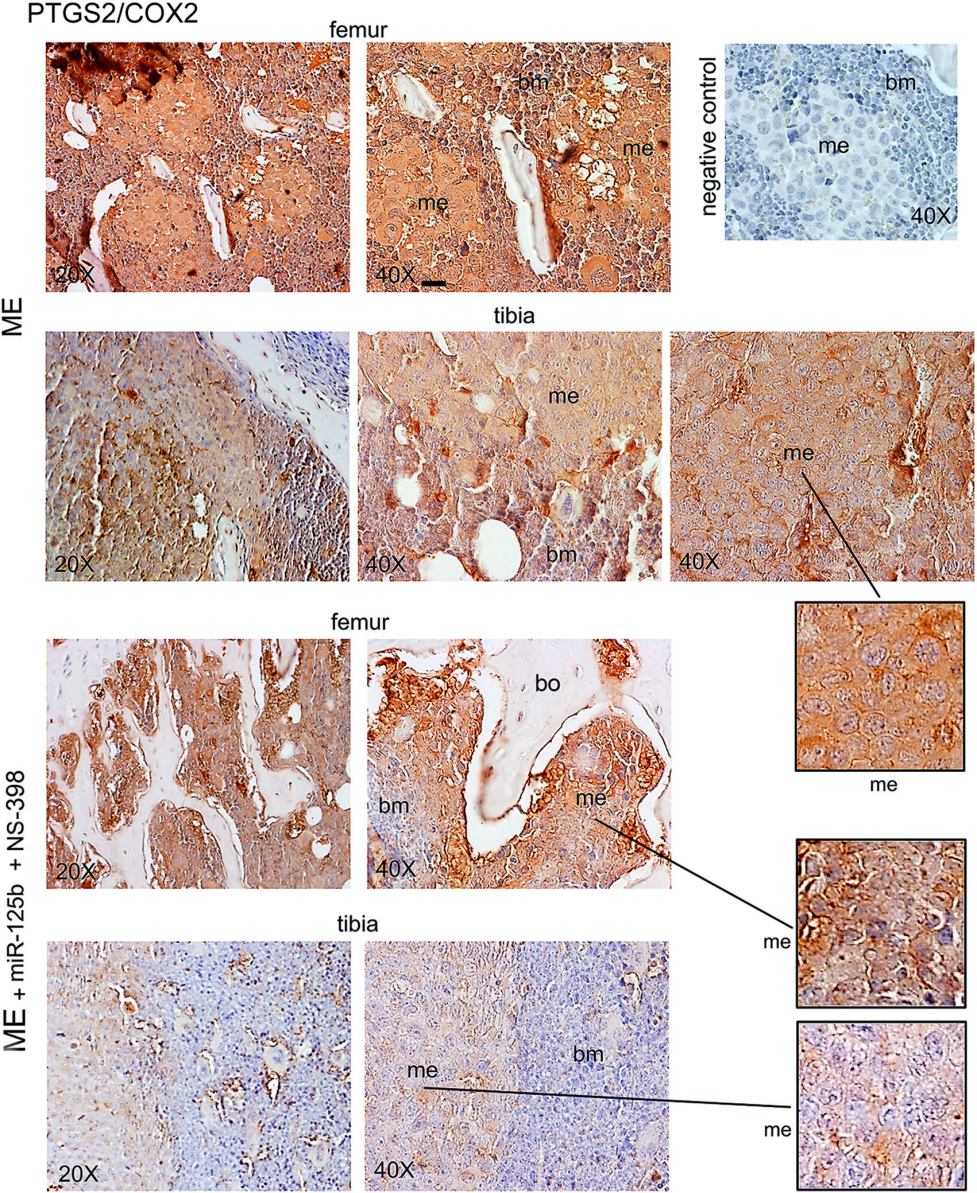
miR-125b in combination with NS-398 affected PTGS2 expression in bone metastasis of xenograft mice. We show representative images of PTGS2 in bone metastasis of mice treated with the combination miR-125b plus NS-398, or not (ME). Serial sections for each specimen from three mice at 26 days were analyzed obtaining similar results. me, metastasis; bm, bone marrow; bo, bone. Scale bar = 120 μm (reported in an exemplificative panel). The negative control was performed without the specific antibody

Immunohistochemistry images of the genes examined are shown in Figs. 3–5. As reported in Fig. 3 and in Supplementary Figure S2, the expression of ETS1 was very high in bone-metastasis both at cytosol and nuclear levels (inset). ETS1 was elevated also in the cytosol of supportive cells of the bone marrow, while in the normal bone the ETS1 signal was scarce (Supplementary Figure S2). The miR-125b plus NS-398 treatment reduced mostly metastatic ETS1, including the nuclear signal (Fig. 3, insets). As shown in Fig. 4, in bone metastases under the combined treatment, the expression of HIF1A largely diminished at cytosol and nuclear levels (insets); the signal of HIF1A extensively decreased also in the cytosol of the bone marrow supportive cells. PTGS2 signal in bone metastases was mild, showing prevalent cytosolic localization, and it was low in the bone marrow: the combined treatment almost completely reduced the signal in the two compartments (Fig. 5). Negative controls did not show specific signals (Figs. 3–5).

The effect of miR-125b or NS-398 treatment on ETS1, HIF1A and PTGS2 is reported in Supplementary Figures S3-S5.

### In 1833 cells miR-125b mimic affected gene expression under the influence of hypoxia or HGF

**Fig. 6:**
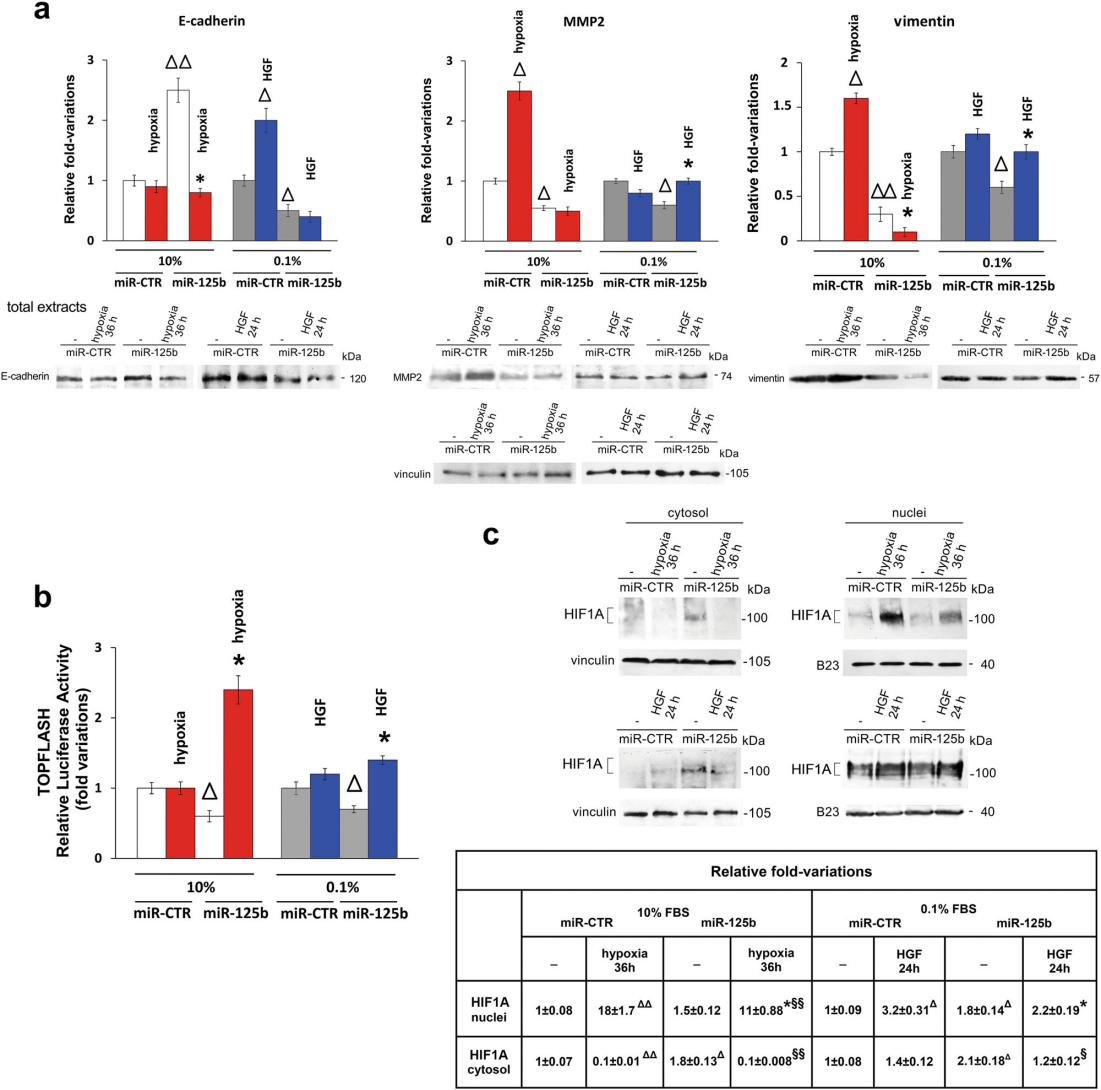
Effect of miR-125b on epithelial and mesenchymal markers and on HIF1A expression under hypoxia or HGF in 1833 cells. **a** Histograms of Western blots densitometric data; the experiments were repeated three times and the means ± S.E. are shown. Vinculin was used for normalization. ^Δ^*P* < 0.05, ^ΔΔ^*P* < 0.005 vs. miR-CTR; *P < 0.05 vs. miR-125b. Also, we report the representative images of the Western blots. **b** TOPFLASH luciferase activity is shown. The assays were performed in triplicate, and were repeated three times; the data are shown as the means ± S.E. ^Δ^*P* < 0.05 vs. miR-CTR; *P < 0.05 vs. miR-125b. **c** Representative images of Western blots performed in triplicate are shown. Vinculin and B23 were used for normalization. The data of densitometric analysis are reported in the table as fold-variations vs. the first lane, considered 1. ^Δ^*P* < 0.05, ^ΔΔ^*P* < 0.005 vs. miR-CTR; *P < 0.05 vs. miR-CTR plus hypoxia or plus HGF; ^§^*P* < 0.05, ^§§^*P* < 0.005 vs. miR-125b

**Fig. 7:**
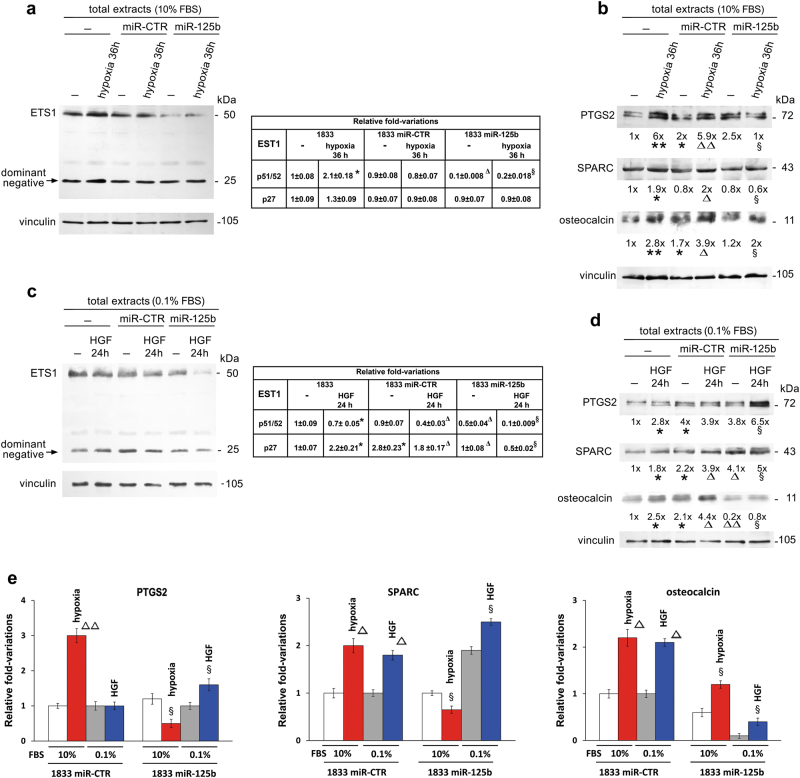
A gene pattern for adhesion/invasion of 1833-bone metastatic cells was affected by miR-125b under hypoxia, or HGF treatment. **a** Representative images of Western blots repeated three times are shown. Vinculin was used for normalization. The data of densitometric analysis are reported in the table as fold-variations vs. the first lane, considered 1. *P < 0.05 vs. untreated 1833 cells; ^Δ^*P* < 0.05 vs. miR-CTR; ^§^*P* < 0.05 vs. miR-CTR under hypoxia. **b** Representative images of Western blots repeated three times are shown. Vinculin was used for normalization. The numbers at the bottom indicate the fold-variations vs. the first lane, considered 1. *P < 0.05, **P < 0.005 vs. untreated 1833 cells; ^Δ^*P* < 0.05, ^ΔΔ^*P* < 0.005 vs. miR-CTR; ^§^*P* < 0.05 vs. miR-CTR under hypoxia. **c** Representative images of Western blots repeated three times are shown. Vinculin was used for normalization. The data of densitometric analysis are reported in the table as fold-variations vs. the first lane, considered 1. *P < 0.05 vs. untreated 1833 cells; ^Δ^*P* < 0.05 vs. miR-CTR; ^§^*P* < 0.05 vs. miR-CTR under HGF. **d** Representative images of Western blots repeated three times are shown. Vinculin was used for normalization. The numbers at the bottom indicate the fold-variations vs. the first lane, considered 1. *P < 0.05 vs. untreated 1833 cells; ^Δ^*P* < 0.05, ^ΔΔ^*P* < 0.005 vs. miR-CTR; ^§^*P* < 0.05 vs. miR-CTR under HGF. **e** To make a comparative evaluation of the gene effects of miR-125b under hypoxia or HGF, we show the histograms of the densitometric values of the Western blots for PTGS2, SPARC and osteocalcin. The data are the means ± S.E. of three separate experiments. ^Δ^*P* < 0.05, ^ΔΔ^*P* < 0.005 vs. respective miR-CTR transfected cells; ^§^*P* < 0.05 vs. respective miR-125b transfected cells

In vitro experiments were performed to better understand the molecular mechanisms underlying miR-125b effects in the bone metastasis xenograft model, and to clarify whether microenvironmental stimuli might play a key regulatory role. To this purpose, in 1833 cells transfected with miR-125b and exposed to hypoxia or HGF, we monitored epithelial, mesenchymal and stem cell markers^7,18^, i.e., E-cadherin, MMP2, vimentin and WNT-pathway activity (Fig. 6). Also, we examined the expression of PTGS2, ETS1 and HIF1A, direct or indirect target genes of miR-125b, as well as of Secreted acidic and rich in cysteine (SPARC) and of osteocalcin, which seem involved in osteoblastic niche (Figs. 6 and 7)^33,34^. Since we have recently shown that ETS1 expression vector affects HIF1A transactivation^22^, miR-125b targeting ETS1 might influence HIF1A expression.

miR-125b mimic augmented the E-cadherin expression only in the presence of 10% fetal bovine serum (FBS): this effect was counteracted by the concomitant exposure to hypoxia (Fig. 6a). Differently, HGF enhanced E-cadherin in 1833 cells transfected with miR-CTR, and miR-125b reduced E-cadherin under basal and HGF conditions. Hypoxia induced MMP2 and vimentin, while being prevented by concomitant miR-125b transfection. Also, miR-125b showed inhibitory effects on MMP2 and vimentin under 0.1% FBS, and HGF caused a reversion (Fig. 6a).

TOPFLASH-gene reporter was used to evaluate WNT pathway activity^35^ (Fig. 6b). miR-125b alone inhibited of about 40% the luciferase activity, while in the presence of hypoxia or HGF a reversion of TOPFLASH activity was observed, peaking (2.5-fold) under hypoxia.

In the nuclei of the cells transfected with miR-CTR, the long-time exposure to hypoxia gave a huge HIF1A increase, and HGF tripled HIF1A: these increases were partly prevented by the concomitant miR-125b treatment. The transfection of miR-125b under 10% FBS (upper panel) and 0.1% FBS (lower panel) doubled HIF1A in the cytosol (Fig. 6c).

Altogether, the 1833 cells required the serum presence for the miR-125b-dependent stimulation of the epithelial marker E-cadherin, while the miR-125b negative effects towards the mesenchymal markers MMP2 and vimentin, the staminal WNT pathway and nuclear HIF1A occurred both under 10 and 0.1% FBS. Also, the reduction of nuclear HIF1A by miR-125b in the presence of hypoxia or HGF, was consistent with the immunohistochemistry findings (Supplementary Figure S4).

Figure 7a shows that 36-h hypoxia doubled the ETS1 protein level in 1833 cells, but not when miR-CTR was transfected. miR-125b in the presence or the absence of hypoxia down-regulated ETS1 (p51/52), while the p27 form of ETS1 was unchanged by the treatments tested.

PTGS2, SPARC and osteocalcin were induced by hypoxia in 1833 cells transfected or not with miR-CTR: miR-125b largely prevented the stimulatory effects of hypoxia (Fig. 7b). The consensus sequences for transcription factors in osteocalcin and SPARC promoters are reported in Supplementary Figure S6: the numerous ETS1 and Runx2 binding sites shown might explain the efficacy of the treatments affecting metastasis phenotype. Putative binding sites of Twist and Snail were also present in the two gene promoters, suggesting a relationship between the changes of gene expression and the metastatic phenotype.

miR-125b transfection, in the presence or the absence of HGF, down-regulated both the p51/52 and the p27 forms of ETS1. In 1833 cells, HGF induced the dominant negative p27, which might interfere in ETS1 activity (Fig. 7c).

The pattern of ETS1 under HGF or hypoxia in vitro was in agreement with that observed in vivo, indicating the important role played by these microenvironmental stimuli in the inhibitory effect of miR-125b towards ETS1 in the xenograft model (Supplementary Figure S3). Only under HGF but not in hypoxic conditions the ETS1-p27 form decreased when the 1833 cells were transfected with miR-CTR or miR-125b.

PTGS2 and SPARC were induced by miR-125b plus HGF; osteocalcin was strongly down-regulated by starvation, and incremented under miR-125b plus HGF (Fig. 7d).

In conclusion, in the histograms (Fig. 7e) we compared the effects of miR-125b under the two microenvironmental stimuli on PTGS2, SPARC and osteocalcin. SPARC is a matricellular protein, not really an ECM component, which might participate in the stiffness and the release of growth factors^36^, initiating changes in neoplastic cell phenotype with an involvement of miRNAs^37^. Osteocalcin is important for the osteoblastic mineralization of endosteal-like ECM: the endosteum lines the inner wall of long bones surrounding the bone marrow cavity, and drives the cellular changes for osteolytic metastases^4,33^. Normally, osteocalcin is produced exclusively by osteoblasts, acting in the bone matrix as a non-collagenous protein, and is now considered an inhibitor of bone mineralization even if opposite hypotheses cannot be excluded^38^. By considering PTGS2 and SPARC, miR-125b was stimulatory in the presence of HGF and inhibitory under hypoxia. Osteocalcin seemed strongly hampered by miR-125b, mostly under starvation, with a partial reversion both under HGF and hypoxia. Thus, the effect of miR-125b on target genes seemed to depend on microenvironmental stimuli.

## Discussion

In the 1833 xenograft model of skeleton metastasis from breast carcinoma the concomitant exposure to miR-125b mimic and NS-398, a specific PTGS2 chemical inhibitor, reduced and delayed the outgrowth of osteolytic bone metastases prolonging mice survival in respect to the single treatments. The miR-125b strongly enhanced the life-protective effect of NS-398, and we hypothesize that the efficacy of the combined treatment depended on the reduction of chemoresistance, known to be associated with EMT^7,18^. The endpoint of our study was to demonstrate that miR-125b enhances the epithelial-like transition in vitro, and that the paracrine stimuli and physical conditions might counteract or favor the phenotypic changes, explaining also the in vivo findings on growth and gene expression in bone metastases.

Importantly, the adverse effect of miR-125b mimic plus NS-398 on metastatic growth started at 4 days from 1833/TGL-cell injection and became highly significant at 9 days, leading to suppose that the hampering of colonization was caused by molecular and cellular modifications at early stages after bone engraftment of DTC. The therapeutic approach consisting in the overexpression of miR-125b which targets a wide spectrum of genes, such as ETS1 and PTGS2, will eventually impact clinical practice preventing bone resorption. The exit from quiescence/dormancy depends on osteoclast activation, and also on stroma composition and neoangiogenic vessels^4^.

**Fig. 8:**
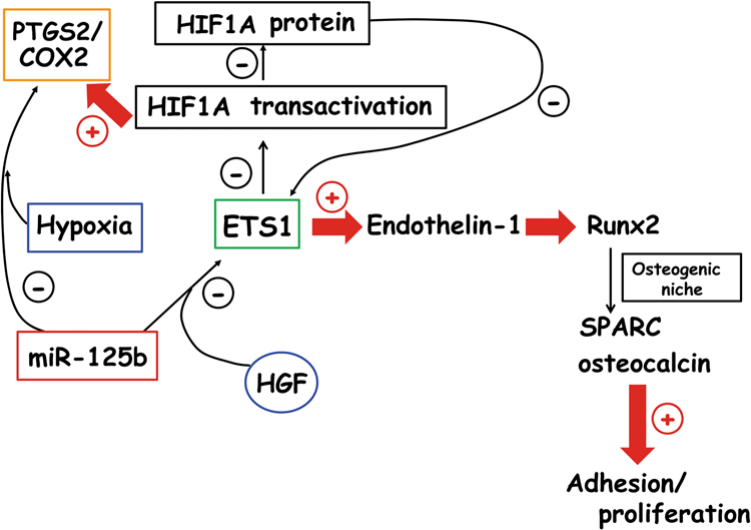
Effects of miR-125b on gene expression under microenvironmental stimuli in bone metastasis

The molecular mechanisms underlying the therapeutic efficacy of the studied treatment miR-125b plus NS-398 would be multiple (Fig. 8). First, the blockade of the autoregulatory loop between ETS1 and HIF1A/HIF1, because of the reciprocal control of these transcription factor activities^22^, and second a complete inhibition of PTGS2 by the concomitant chemical and miR-125b treatment. At support, PTGS2 signal remarkably decreased in osseous metastases and the bone marrow, also due to the down-regulation of HIF1A throughout the metastatic tissue: the alpha/beta heterodimer HIF1 is a transcription factor known to target PTGS2 and ETS1^20,25^. Our proposed therapy would have a direct effect on PTGS2, through miR-125b action on HuR sites, even if a post-transcriptional effect of miR-125b on ETS1 cannot be excluded. Also, an indirect effect of the therapy on ETS1 through HIF1A downregulation might occur, consistent with the decreases of ETS1 and HIF1A in cytosol and nuclei of metastases and in the bone marrow. We suggest that these amplifying effects might impinge on invasion and angiogenesis, because ETS1 and PTGS2 are key players in these processes: PTGS2 through prostaglandin E2 regulates HIF1A nuclear translocation and HIF1 activity (Supplementary Figure S4)^20,25^. Capillaries are associated with quiescent and actively resorbing endosteal surfaces, and are implicated in development of bone metastasis, permitting the access of DTC to bone-remodeling compartment or to the immediate adjacent microenvironment^4^.

Third, in vivo phenotypic changes under miR-125b plus NS-398 seemed to depend on the metastatic down-regulations of ETS1 and HIF1A, which are critical for the invasive phenotype and staminality^20,39^. Also, in agreement with the hypothesis that in 1833-xenograft model miR-125b decreased the metastasis resistance to chemotherapy, the microenvironmental stimuli would be critical for the effect of miR-125b in vivo, suggesting that the bone context encountered by DTC would be fundamental for their fate. The skeleton microenvironment is modified by the engraftment of metastatic cells influencing the EMT^18,37^, and it would interfere in the therapies such as that with miR-125b mimic.

The axis HGF/Met receptor seems to be implicated in stroma-induced drug desensitization^20^, and hypoxia conferring stem like properties might give resistance to therapy^39^. Consistently, we found that the tumor suppressor gene miR-125b in 1833 cells reduced WNT activity and targeted transcription factors, such as ETS1, HIF1 and WNT, likely affecting gene expression in bone metastasis. Numerous ETS1 consensus sequences are present in SPARC and osteocalcin promoters (Supplementary Figure S6). In addition, considering that in bone-metastatic cells miR-125b enhanced the expression of E-cadherin, while decreasing MMP2 and vimentin, our present results suggested a switch towards an epithelial phenotype, which is known to be more responsive to chemotherapy than the mesenchymal/invasive phenotype^7,18,20^.

In bone metastatic cells, which show an epithelial phenotype^30^, miR-125b might antagonize the function of miR-200^40^: we propose that miR-125b by blocking p53 prevents miR-200-inhibitory function^11,19,37^. As a consequence, Twist activity might mediate the MET metastable phenotype with E-cadherin expression^9^. The miR-125b effect on the gene expression of bone metastatic cells depended on serum conditions, HGF and hypoxia exposure, favoring the reduction of ETS1 expression in vitro, while the microenvironmental stimuli counteracted the miR-125b-dependent epithelial phenotype with E-cadherin reduction by hypoxia and MMP2 and vimentin enhancements by HGF.

Altogether, the knowledge of phenotypic changes under therapy were further deepened by studying SPARC and osteocalcin, which are target genes of Runx2 transcription factor^34,41^; HIF1A expression is also controlled by Runx2 by preventing the ubiquitination^42^. Runx2 plays key roles in bone metastasis from breast carcinoma^43,44^, being regulated by Endothelin-1, HGF and hypoxia^35,45^. Notably, bone-metastatic cells acquire osteomimicry by producing SPARC and osteocalcin, as shown in the 1833-xenograft model^32,34,46^.

HGF and hypoxia caused SPARC accumulation, which might be important to determine the adhesive properties of bone metastasis in the osteoblastic niche, ECM stiffness and growth-factor release, and miR-125b counteracted the stimulatory effect of hypoxia. Therefore, the miR-125b mimic might reduce bone metastasis colonization in vivo influencing SPARC roles in metastasis plasticity and in metastasis dormancy, by the regulation of biological and physical properties of the ECM^34,36,46,47^. HIF1A beyond SPARC participates in metastasis dormancy^48^.

In conclusion, a strong down-regulation of osteocalcin was observed in the presence of miR-125b under serum deprivation, indicating the susceptibility to growth factors, while HGF restoration not only reversed osteocalcin expression, but also increased PTGS2 and SPARC under miR-125b. The miR-125b-dependent impairment of osteocalcin, considered an inhibitor of mineralization^38^, might be related to protection from osteolysis: this effect would be partly prevented by the microenvironmental stimuli. The combined therapeutic approach miR-125b plus NS-398 seems promising for the early hampering effects on metastatic growth and osteolytic lesions, considering also that the initial metastatic steps and therapy efficacy are strongly influenced by the microenvironment conditions.

## Materials and Methods

### Cell transfection

The 1833 bone metastatic clone, derived from MDA-MB-231 breast adenocarcinoma cells, and the 1833 cells retrovirally transfected with HSV1-tk/GFP/firefly luciferase construct (1833/TGL)^32^, were a gift from Dr. J. Massagué (Memorial Sloan-Kettering Cancer Center, New York, NY, USA).

The cells, routinely maintained in DMEM containing 10% FBS (Sigma-Aldrich, Saint Louis, MO), were used after two or three passages in culture. The 1833 and 1833/TGL cells were infected with miR-125b expression lentivirus (hsa-miR-125b-5p MIMAT0000423, GenTarget Inc., San Diego, CA, USA) or with miR-CTR (negative control, empty miR lentivirus, GenTarget Inc.), following the manufacturer’s recommended procedures. Lentiviral vectors co-expressed puromycin resistance for the selection of infected cells. A pilot experiment has been done to determine the antibiotic’s kill curve for our cells. Thus, 72-h infected cells were selected with 2 μg/ml puromycin, and were maintained under selection in all the experiments.

In another group of experiments, flasks containing 1833 cells at 70% of confluence were used to transfect the TOPFLASH gene reporter from Dr. B.M. Gumbiner (Memorial Sloan-Kettering Cancer Center, New York, NY, USA). For the transfection of the TOPFLASH construct, we performed an incubation mixture 3:1, DNA:Fugene 6 (Roche Diagnostics, Monza, Italy); the mixture contained also the internal control pRL-TK (*Renilla* Luciferase Plasmid). Firefly/*Renilla* luciferase activity ratios were calculated by the software^9^.

### Animal treatment

Animal studies were carried out according to the Institutional Guide for Care and Use of Laboratory Animals, and the Directive 2010/63/UE. All mice were bred and maintained under specific pathogen-free conditions in the institutional animal facility of the Scientific Institute San Raffaele, Milano, Italy. All experiments were performed with the protocol approved by the Institutional Animal Care and Use Committee of Scientific Institute San Raffaele and by Ministero della Salute. Anaesthetized nu/nu mice were treated as follows: one animal group (ME, *n* = 11) was injected with the 1833/TGL cells; a second group (ME + miR-125b, *n* = 11) received 1833/TGL cells infected with miR-125b; a third group (ME + miR-125b + NS-398, *n* = 10) received 1833/TGL cells infected with miR-125b, and the mice were concomitantly injected with 20 mg/kg NS-398 i.p. 5 days/week until the suppression; the fourth group was treated only with NS-398 (*n* = 10); the fifth group was administered 1833/TGL cells infected miR-neg-CTR (ME + miR-CTR, *n* = 4). For the xenograft model preparation, 5 × 10^5^ infected or non-infected cells were injected to the mice, that were monitored by Optical imaging and μCT assay^6^, and were sacrificed to prevent the suffering. Three animals per group were sacrificed at the indicated times to perform immunohistochemistry and Hematoxylin & Eosin assays.

The injection efficiency for all the mice was controlled by monitoring the bioluminescence signal 1 h after xenografting. We normalized the data of bioluminescence for each animal with the value obtained at 24 h. This procedure was made to exclude a potential impact of the pretreatments, i.e., miR-125b-cell transfection or NS-398 exposure, on extravasation and homing with an interference in the evaluation of metastasis growth.

### Immunohistochemistry

Bone samples fixed with 10% formalin, were decalcified, embedded in paraffin, and serial sections were prepared^49^. To evaluate ETS1, HIF1A and PTGS2, the immunostaining was performed with anti-Ets-1 (1:50, C20 Santa Cruz Biotechnology, Santa Cruz, CA, USA), anti-HIF-1α (1:100, Novus Biologicals, Littleton, CO, USA), and anti-COX2 (1:50, Cayman Chemical, Ann Arbor, MI, USA).

### Western blot assay

Some flasks containing 1833 cells infected with miR-125b mimic were exposed to hypoxia^50^, or to 100 ng/ml HGF under starvation^9^, and total and nuclear protein extracts were prepared. To evaluate ETS1, HIF1A and PTGS2, immunoblots were performed with anti-Ets-1 (1:2000), anti-HIF-1α (clone54) (1:350, BD-Transduction Laboratories, Franklin Lakes, NJ, USA), and anti-COX2 (1:100). Also, anti-E-cadherin (1:1000, clone 36 Transduction Laboratories, Bedford, MA, USA), anti-MMP2 (1:500, Novus Biologicals, Abingdon, UK), anti-vimentin (1:500 Santa-Cruz Biotechnology), anti-SPARC (1:200, H-90 Santa Cruz Biotechnology), anti-osteocalcin (5 μg/ml, Abcam, Cambridge, UK), anti-B23 (1:1000, H106 Santa Cruz Biotechnology), or anti-vinculin (1:1000, Cell Signaling, Leiden, The Netherlands) was used for immunoblotting. Densitometric analysis was performed after reaction with ECL plus chemiluminescence kit from Thermo-Fisher Scientific (Waltham, MA, USA) or Clarity Max Western ECL Substrate-Luminol Solution (Biorad, Hercules, CA, USA).

### Statistical analysis

The statistical analysis of the values of bioluminescence for the xenograft mice groups, as well as of Western blots densitometry and luciferase activity values was performed by analysis of variance. The number of independent experiments has been indicated in the Legends of the Figures. The data are shown as means ± S.E., and their significance was evaluated on the original values in the case of fold-variations. *P* < 0.05 was considered significant. For xenograft mice, the survival data were analyzed by Kaplan–Meier method and the Log-rank (Mantel–Cox) test. *P* < 0.05 was considered significant.

## Electronic supplementary material

Supplementary Information

Supp. Fig. 1

Supp. Fig. 2

Supp. Fig. 3

Suppl. Fig. 4

Suppl. Fig. 5

Suppl. Fig. 6
