# Supplementary material for: The therapeutic effect of miR-125b is enhanced by the prostaglandin endoperoxide synthase 2/cyclooxygenase 2 blockade and hampers ETS1 in the context of the microenvironment of bone metastasis
Source: Cell Death Dis. 2018 Apr 27;9(5):472. doi: 10.1038/s41419-018-0499-8 (PMC5920088; doi:10.1038/s41419-018-0499-8)
Supplement: Supplementary file 1 — Supplementary Information [file 41419_2018_499_MOESM1_ESM.docx]

**SUPPLEMENTARY INFORMATION**

**Supplementary Materials and Methods**

**Supplementary Figure Legends**

**Supplementary References**

**Supplementary Materials and Methods**

**Cell culture**

We used 1833 cells, which is a clone derived from MDA-MB231 breast adenocarcinoma cells. These cell lines were compared for the transcriptomic profile identifying a gene set whose expression pattern is associated with, and promotes the formation of, metastasis to bone,^1^ and they were authenticated with the method of short-tandem repeat profiling (STR) of nine highly polymorphic STR loci plus amelogenin on September 2014 (Cell Service from IRCCS-Azienda Ospedaliera Universitaria San Martino-IST-Istituto Nazionale per la Ricerca sul Cancro, Genova, Italy). The 1833 cells, routinely maintained in DMEM containing 10% FBS (Sigma-Aldrich, Saint Louis, MO), were used after 2 or 3 passages in culture.

**Intracardiac cell injection, Optical Imaging analysis and μCT image acquisition**

The 1833/TGL cells were harvested from subconfluent cultures, washed and resuspended in PBS. Cell aliquots (5x10^5^ cells/0.1 ml) were injected into the left cardiac ventricle of 4-week-old female nu/nu mice (Envigo, Italy), using 26G needles.^2^ For cell injection the mice were anesthetized with Avertin (0.2 ml/10 g body weight). A successful injection was characterized by the pumping of arterial blood into the syringe. At early times after xenografting, we verified by Optical Imaging the presence of bioluminescent circulating cells in the mice. Acquisition time for bioluminescence was 5 min at the beginning of observation time points (1 and 24 h); for the following observations, the acquisition time was reduced to 1 min in accordance with the enhanced signal strength, and to avoid signal saturation. Ventral and dorsal projections were examined. For normalization of the data, regions of interest all over the skeleton (ROI) were defined, and the corresponding bioluminescence values at 24 h were evaluated in the five groups of xenograft mice. We verified that all the groups of xenograft mice had similar bioluminescence value at ROI level at 24 h, and the following bioluminescence signals for each animal were normalized to the value obtained at this time: the procedure avoids the consideration of variations due to homing and extravasation.

To monitor bioluminescence, Firefly D-luciferin (150 mg/kg i.p.) was given under anesthesia; metastasis outgrowth was evaluated by Optical Imaging, using IVIS Spectrum CT System (Perkin Elmer, Milano, Italy), and photon-emission was quantified with Living-Image 4.5 Software (Perkin Elmer). The same apparatus was used to evaluate osteolysis by micro-computed tomography (µCT); three dimensional images were reconstructed using the software (San Raffaele Institute, Medical Physics Department and Centre for Experimental Imaging). μCT images were acquired using the dedicated X-ray module of the IVIS Spectrum CT System with the following parameters: X-ray tube voltage= 50 kV, current = 1 mA, number of views= 720, voxel size = 0.075 mm^3^.

**Supplementary Figure Legends**

**Supplementary Figure S1 Effect of miR-125b combined with NS-398 on bone metastasis growth.** (**a**) Hind-limb bones form xenograft mice, injected with 1833/TGL cells transfected with miR-125b and concomitantly exposed to NS-398, were collected at 26 and 29 days, and were processed to prepare serial sections, which were stained with Hematoxylin & Eosin. Representative images are shown for bone metastasis of treated and untreated xenograft mice, n=3. A reduction of metastasis wideness of about 50% was observed in xenograft mice 26 days after the combined treatment, compared with ME (untreated xenograft model of bone metastasis). At 29 days, osteolytic metastases incremented in wideness mostly in the femur of the mice injected with 1833/TGL cells transduced with miR-125b and concomitantly exposed to NS-398. (**b**) Bioluminescence signal was evaluated by Optical Imaging. The combined treatment miR-125b plus NS398 reduced the bioluminescence of about 60% at 26 days, in respect to the values of ME and of the single treatments, *P<0.05.

**Supplementary Figure S2 ETS1 expression in bone from normal mice and in the skeleton metastasis of the xenograft mice.** We show representative images of immunohistochemistry analysis of ETS1 performed with anti-Ets-1 antiboby. We analyzed serial sections for each bone specimen from three control mice, and three xenograft mice. bm, bone marrow; bo, bone; me, metastasis, gp growth plate. Insets show the magnification of the images. Negative controls were performed without the specific antibody, and did not give signals. The supportive cells in the bone marrow of metastasis-bearing mice (panels 5 and 6) were highly positive for ETS1 in respect to those in control bone (panels 1 and 2). A strong ETS1 signal was observed in bone-metastatic cells (panels 3 and 4).

**Supplementary Figure S3 Effect of NS-398 or miR-125b on metastatic ETS1 of xenograft mice.**

We show representative images of immunohistochemistry analysis of ETS1. Serial sections for each specimen from three mice per group were analyzed 26 days after xenografting. me, metastasis; bm, bone marrow; bo, bone (see 40x or 60x panel). miR-125b exposure decreased ETS1 signal in bone metastatic cells and in the bone marrow, compared with untreated bone metastasis of xenograft mice (ME). NS-398 is a specific inhibitor of prostaglandin endoperoxide synthase 2**,** also known as cyclooxygenase 2 (COX2);^3^ it also exerted an inhibitory effect on ETS1 (see table in Figure 2 of the paper).

**Supplementary Figure S4** **Effect of NS-398 or miR-125b on metastatic** **hypoxia inducible factor 1 alpha subunit (HIF1A) of xenograft mice.** We show representative images of immunohistochemistry analysis of HIF-1A, performed with anti-HIF-1α antibody. Serial sections for each specimen from three mice per group were analyzed 26 days after xenografting. me, metastasis; bm, bone marrow; bo, bone (see 40x or 60x panel). NS-398 treatment as well as miR-125b exposure decreased HIF1A signal in cytosol/nuclei: the effect was observed in bone metastatic cells and in the bone marrow, compared with untreated bone metastasis of xenograft mice (ME). This result might be explained considering the role of COX2 products in the nuclear translocation of HIF1A, and HIF1 activation.^3^

The scheme shows that the inhibitory effect of miR-125b under hypoxia on ETS1 might influence HIF1A expression/HIF1 activity and angiogenesis in bone metastasis counteracting the growth. Numerous ETS1 putative binding sites are present in the HIF1A promoter.

**Supplementary Figure S5** **Effect of NS-398 or miR-125b on metastatic COX2 of xenograft mice.** We show representative images of immunohistochemistry analysis of COX2. Serial sections for each specimen from three mice per group were analyzed 26 days after xenografting. me, metastasis; bm, bone marrow; gp, growth plate; bo, bone; mk, megakariocytes (see 40x panel). NS-398 treatment markedly decreased COX2 signal in cytosol/nuclei, and it was more effective than miR-125b exposure. These effects were observed in bone metastatic cells and in the bone marrow, compared with untreated bone metastasis of xenograft mice (ME). The data demonstrated the efficacy of NS-398 treatment in inhibiting COX2.

**Supplementary Figure S6** The principal consensus sequences present in the promoters of SPARC and Osteocalcin are shown. Numerous consensus sites for Runx2 and Twist are present in Osteocalcin promoter, suggesting a critical regulatory role for these transcription factors in Osteocalcin expression.

**Supplementary References**

1. Kang, Y., *et al.* A multigenic program mediating breast cancer metastasis to bone. *Cancer Cell* **3**, 537-549 (2003).

2. Maroni, P., *et al.* Osteolytic bone metastasis is hampered by impinging on the interplay among autophagy, anoikis and ossification. *Cell Death Dis.* **5**, e1005 (2014).

3. Maroni, P., *et al.* Nuclear co-localization and functional interaction of COX-2 and HIF-1α characterize bone metastasis of human breast carcinoma. *Breast Cancer Res. Treat.* **129**, 433-450 (2011).
