# Supplementary figures and images for: The therapeutic effect of miR-125b is enhanced by the prostaglandin endoperoxide synthase 2/cyclooxygenase 2 blockade and hampers ETS1 in the context of the microenvironment of bone metastasis
Source: Cell Death Dis. 2018 Apr 27;9(5):472. doi: 10.1038/s41419-018-0499-8 (PMC5920088; doi:10.1038/s41419-018-0499-8)

**a**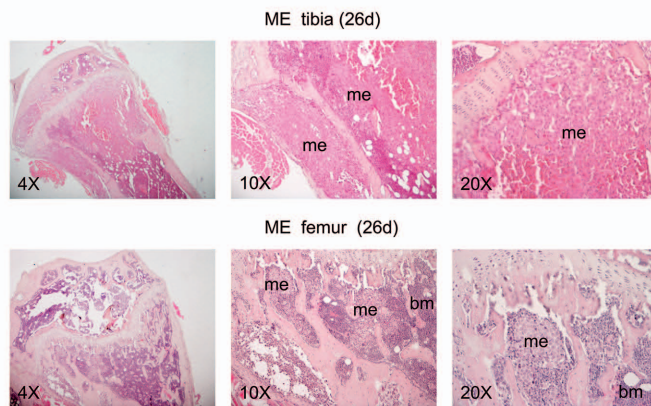**b**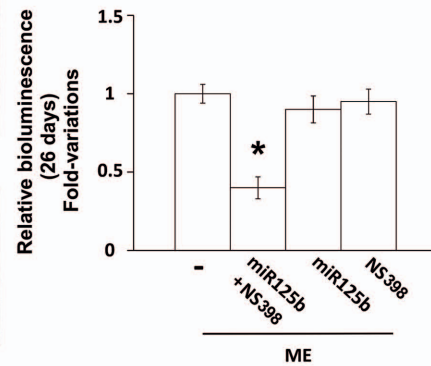

ME + miR125b + NS398 tibia (26d)

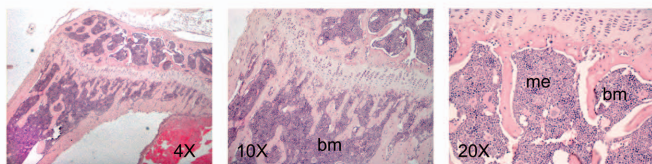

ME + miR125b + NS398 tibia (29d)

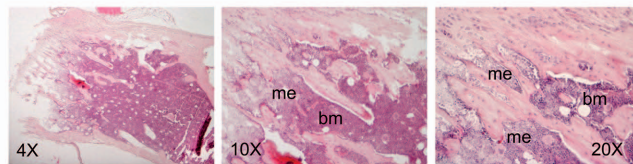

ME + miR125b + NS398 femur (26d)

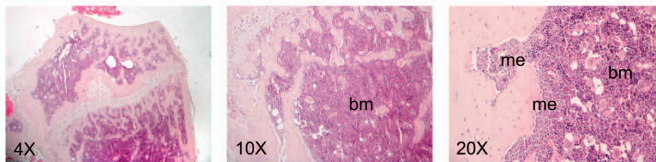

ME + miR125b + NS398 femur (29d)

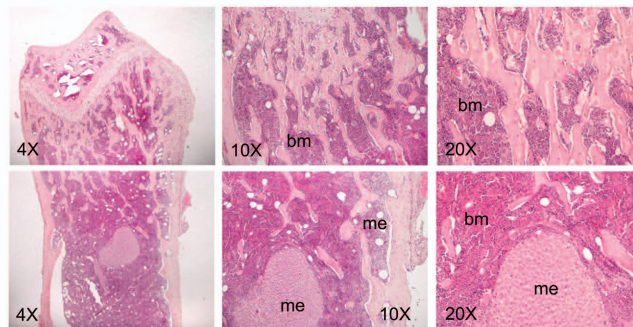

Suppl. Fig. 1

Supplement: Supplementary file 2 — Supp. Fig. 1 [file 41419_2018_499_MOESM2_ESM.pdf]

Ets1

control bone

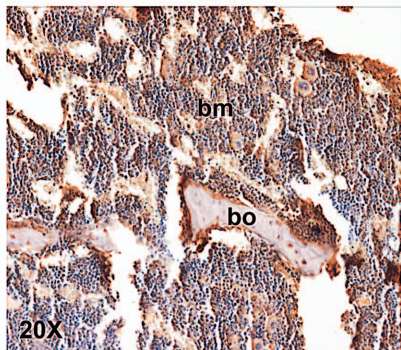

1

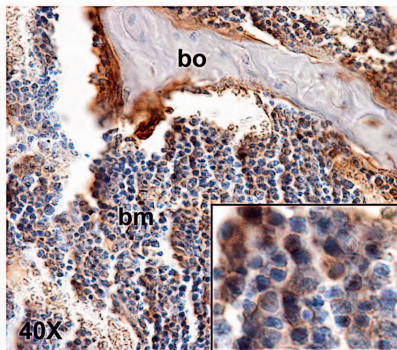

bone metastasis

2

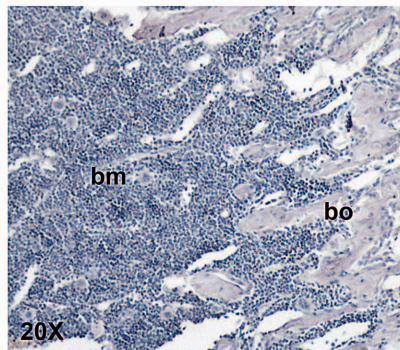

negative control

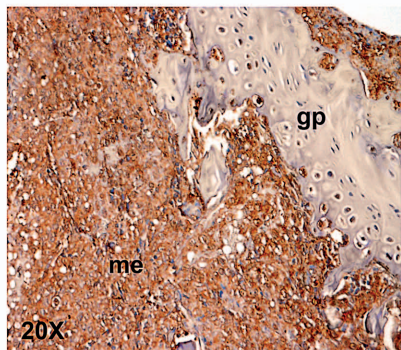

3

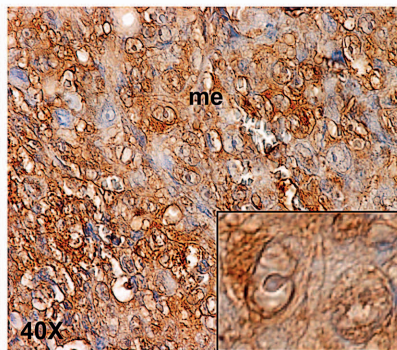

4

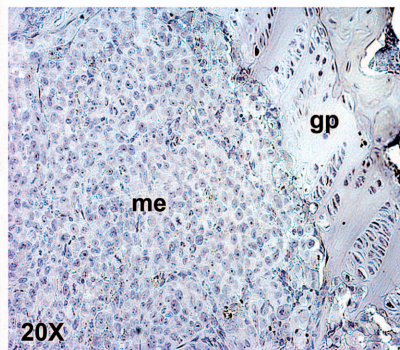

negative control

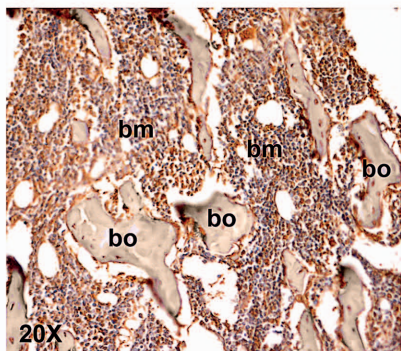

5

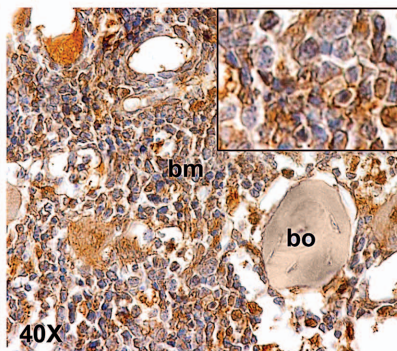

6

Suppl. Fig. 2

Supplement: Supplementary file 3 — Supp. Fig. 2 [file 41419_2018_499_MOESM3_ESM.pdf]

Ets1

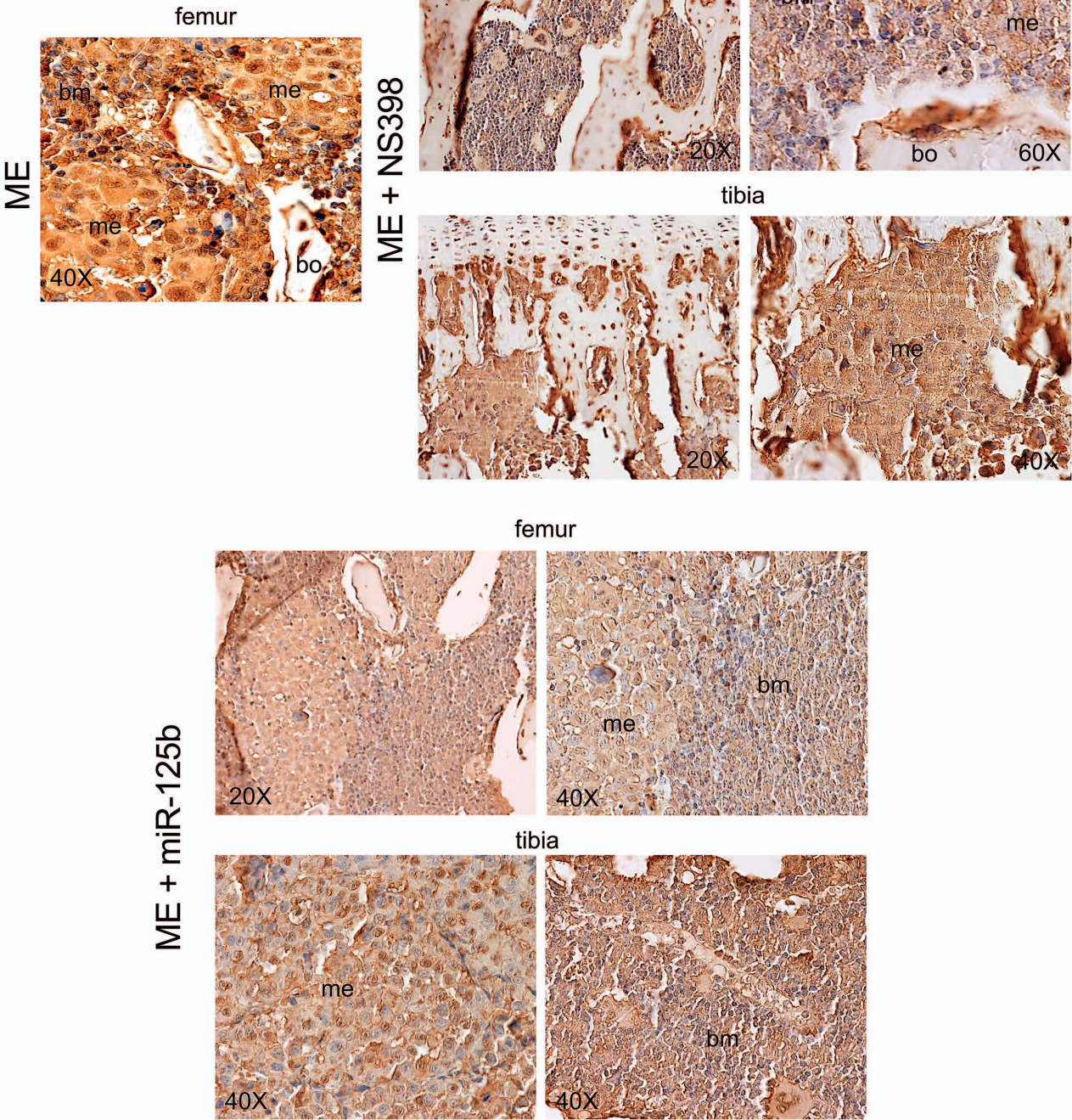

Suppl. Fig. 3

Supplement: Supplementary file 4 — Supp. Fig. 3 [file 41419_2018_499_MOESM4_ESM.pdf]

HIF-1 $\alpha$

ME

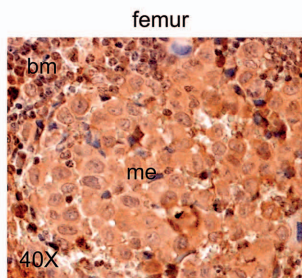

ME + NS398

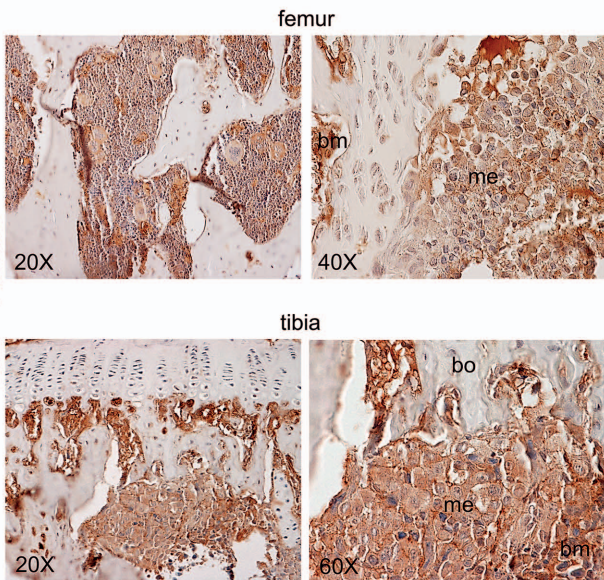

ME + miR-125b

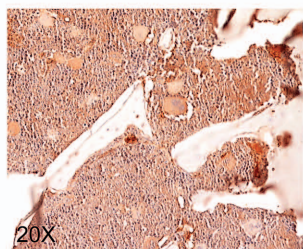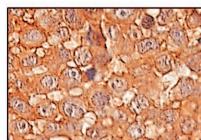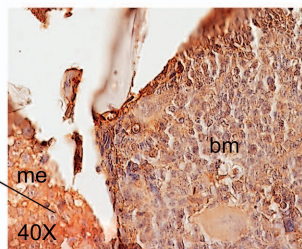

tibia

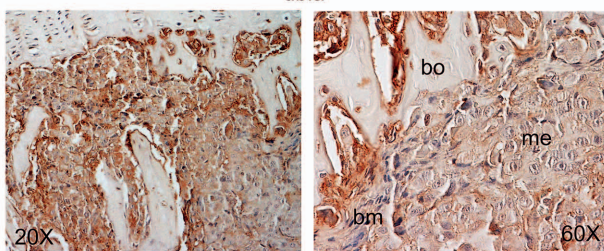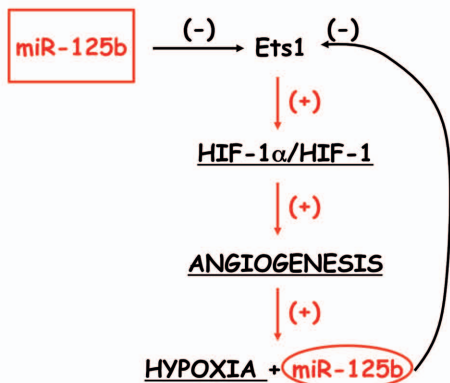

Suppl. Fig. 4

Supplement: Supplementary file 5 — Suppl. Fig. 4 [file 41419_2018_499_MOESM5_ESM.pdf]

# COX2

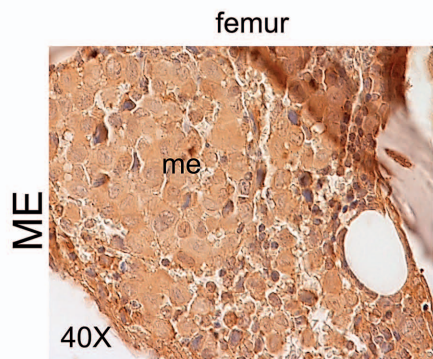

ME + NS398

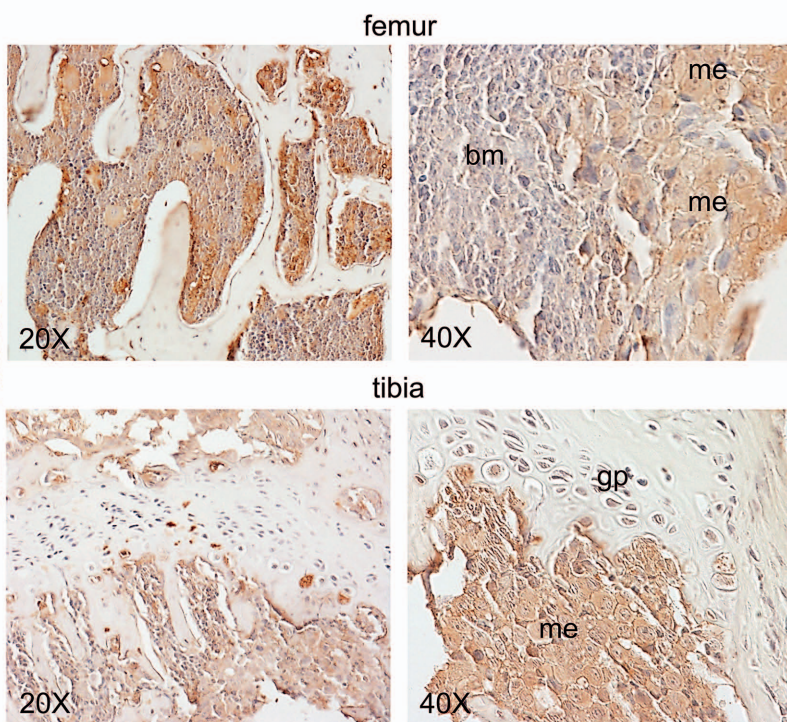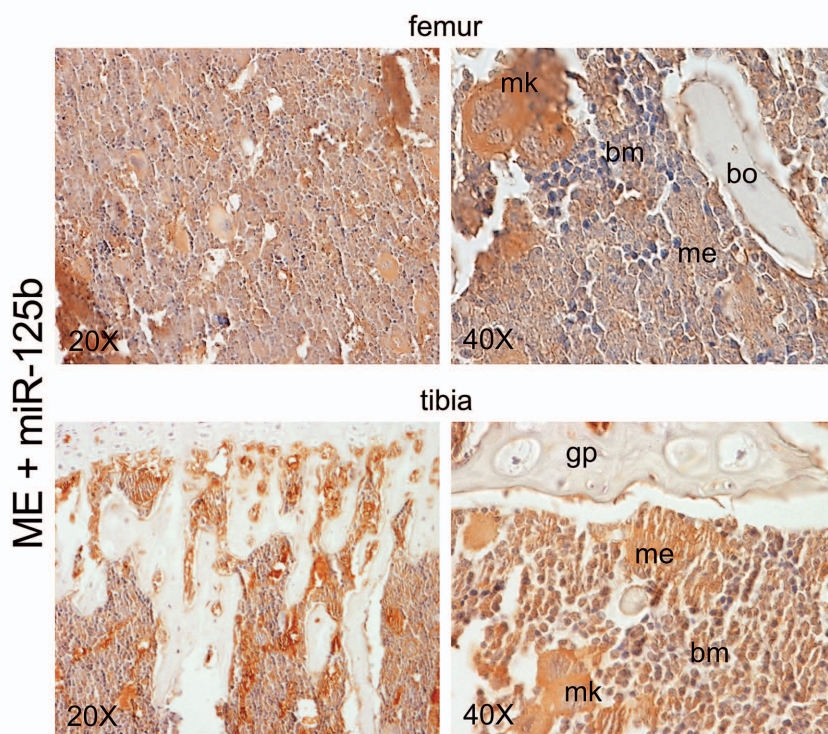

Supplement: Supplementary file 6 — Suppl. Fig. 5 [file 41419_2018_499_MOESM6_ESM.pdf]

# Human SPARC promoter

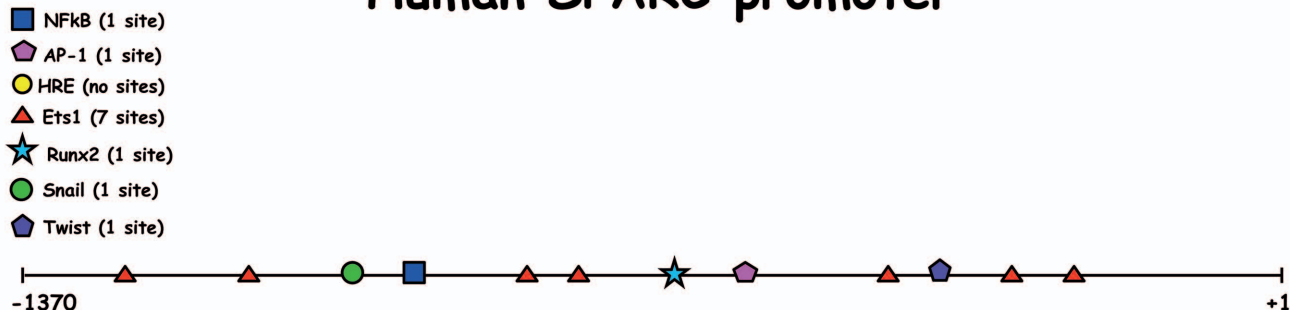

# Human Osteocalcin promoter

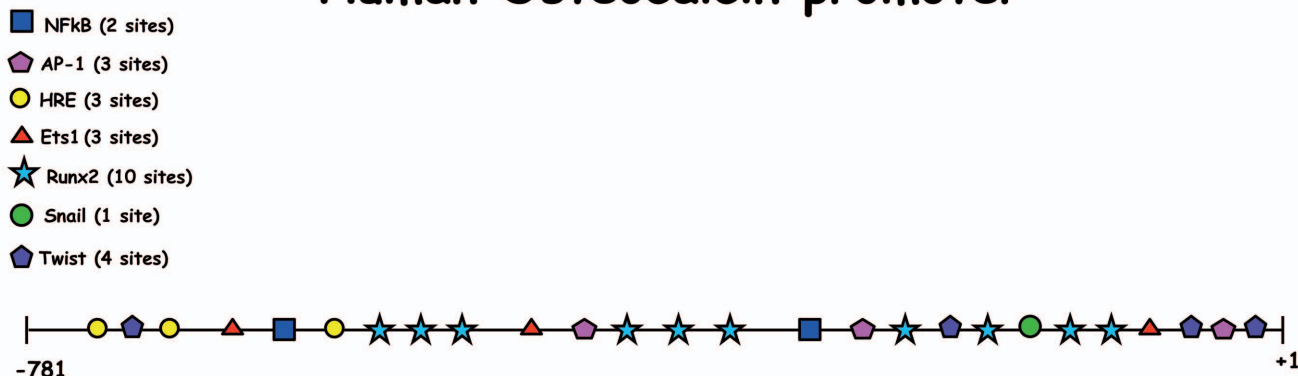

Supplement: Supplementary file 7 — Suppl. Fig. 6 [file 41419_2018_499_MOESM7_ESM.pdf]
